# Collaborative e-Learning Using Streaming Video and Asynchronous Discussion Boards to Teach the Cognitive Foundation of Medical Interviewing: A Case Study

**DOI:** 10.2196/jmir.5.2.e13

**Published:** 2003-06-27

**Authors:** John M Wiecha, Robert Gramling, Phyllis Joachim, Hannelore Vanderschmidt

**Affiliations:** ^1^Boston University School of MedicineBoston Medical CenterBoston MAUSA; ^2^Brown UniversityDepartment of Family MedicineProvidence RIUSA; ^3^Boston University School of MedicineDepartment of Family MedicineBoston MAUSA; ^4^School of Public HealthBoston UniversityCenter of Educational Development in HealthBoston MAUSA

**Keywords:** Education, distance, medical history taking, education, medical, online systems, students, medical, communication, physician-patient relations, clinical competence, Internet, World Wide Web

## Abstract

**Background:**

Advances in electronic technology have created opportunities for new instructional designs of medical curricula.

**Objective:**

We created and evaluated a 4-week online elective course for medical students to teach the cognitive basis for interviewing skills.

**Methods:**

Ten students, from 2 medical schools, studied online modules on interviewing concepts and viewed videos illustrating the concepts. They then participated in asynchronous discussion groups designed to reinforce course concepts, stimulate reflective learning, and promote peer learning.

**Results:**

In qualitative evaluations, learners reported improvements in self-awareness; increased understanding of interviewing concepts; and benefits of online learning vs face to face learning. Participants reported high levels of satisfaction with online learning and with achievement of course objectives. Self-reported knowledge scores increased significantly from pre-course completion to post-course completion.

**Conclusions:**

Online education has significant potential to augment curriculum on the medical interview, particularly among students trained in community settings geographically distant from their academic medical center.

## Introduction

A number of organizations [1- 3] have identified deficiencies in physician communication-skills training. Strengthening instruction in communication skills is a priority national objective for US medical schools [4]. Learning effective communication requires a cognitive foundation of interviewing theories and concepts [4]. A curriculum on communication concepts and strategies should provide understanding of fundamental skills and processes, and will establish a sound foundation for learning skills [4]. Such knowledge objectives have typically been best taught in years one and two of the curriculum [4]. Decentralization [5], a growing emphasis on adult learning principles, and use of distance education requires new thinking about curricular design and delivery. This paper reports our experiences with a new online method for teaching communication concepts to medical students.

### Materials and Methods

**Figure 1:**
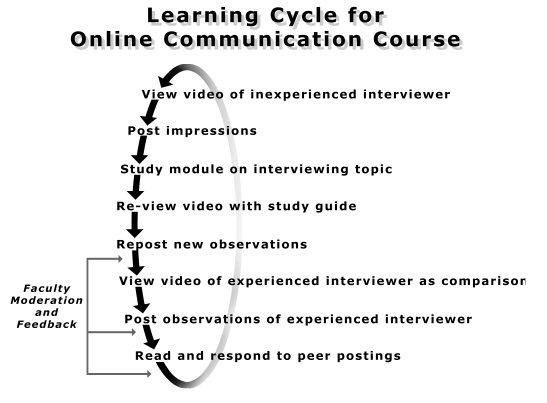
Sequence of educational activities

The instructional design we use for online courses [6] has the learner follow a deliberate sequence of educational activities (Figure 1). Guided by the SEGUE (Set the Stage, Elicit Information, Give Information, Understand the Patient's Perspective, End the Encounter) framework of communication tasks [4], over 4 weeks in an online elective course we consecutively addressed questioning techniques, affect and nonverbal cues, eliciting the cardinal features of a symptom, and stages and transitions.

**Figure 2:**
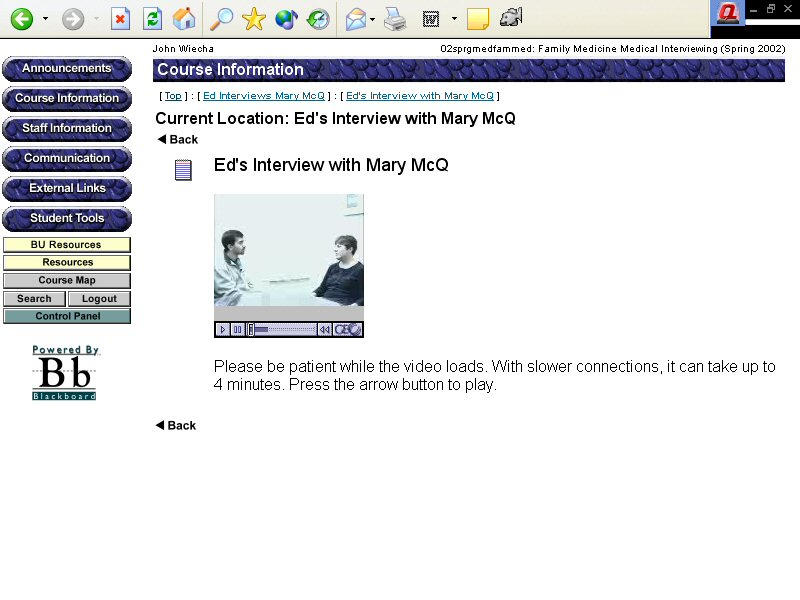
Videos on the Blackboard e-learning site showing inexperienced and experienced interviews with patients

Blackboard, a web-based learning system [7] was used to organize course material and activities (Multimedia Appendix 1). Two videos, delivered online through the Blackboard courseware and produced with GeoSystems compression software, illustrated the concepts presented each week in the Web-based text modules (Figure 2). The videos were between 15 and 20 minutes in length. The first video demonstrated inexperienced interviewing by showing a first-year student interviewing a woman (Mary) with a depressed affect and dyspepsia. The second video was of a family physician interviewing a young man (Ed) with the same symptoms, and demonstrated a more-experienced interviewer. Each video was streamed through the course Web site.

Students received access to a moderated, asynchronous discussion board and were required to post their impressions and observations each week. If necessary (that is, if they lagged in posting), they were reminded by the moderator. Using established principles [8- 9] (including probing participants for deeper reflection, challenging assertions by contrasting differing viewpoints and observations, and summarizing concepts and conclusions included in the postings to provide closure to each week's discussion), trained faculty moderated the discussion groups. Discourse should be a component of courses teaching communication concepts [4]. At the course midpoint (ie, after 2 weeks) and at course end, students also received written feedback on their participation and performance by personal e-mail from one of the authors (PJ).

### Evaluation Instruments and Processes

Qualitative assessments included one-to-one in-person interviews using open-ended questions, analysis of student course postings, and a face-to-face focus group with all 10 students, done by a facilitator previously unknown to the students. Interviews and focus groups were recorded and transcribed, and analyzed for emerging themes by one investigator (RG).

Eleven formative evaluation questions (Table 1) were presented 1 week after the course using a Web-based questionnaire. Each question was scored on a 7-point Likert scale.

Students also completed pre-course and post-course Web-based questionnaires with 21 items (Multimedia Appendix 2 and Multimedia Appendix 3), each scored on a scale from 1 (no understanding) to 10 (complete understanding), grouped into 4 categories (shown in Table 2) corresponding to the major course objectives. Before/after scores were compared using a paired t test.

We calculated for each student a mean score on the baseline knowledge items. This score was linearly correlated with the number of interviews each student reported having completed to date ( *r*= 0.9412, *P*< .001), providing support for the construct validity of the self-reported knowledge measures (Figure 3).

**Table 1:** Student evaluation of the process of learning online

	**Agreement (n = 9)**	
	Mean*(Maximum = 7.0)	**Agree Strongly n (%)**
The faculty interview was effective in demonstrating principles of interviewing	6.4	7 (77.8)
The student interview was effective in demonstrating principles of interviewing	5.1	3 (33.3)
I valued interacting with faculty online via the threaded discussions groups	6.3	6 (66.7)
I valued interacting with other students online via the threaded discussion groups	6.2	5 (55.6)
I received feedback on my questions and concerns from BU faculty during the course	6.1	4 (44.4)
During this course I learned from other students	5.7	1 (11.1)
I had adequate time in my schedule to complete the assignments in the online course	6.1	6 (66.7)
The online course was easy to use	6.3	7 (77.8)
I enjoyed the online course	5.9	3 (33.3)
I would recommend that other students take this course	6.1	5 (55.6)
I would be interested in other online courses in medical school	6.1	5 (55.6)

^*^ Scaled as: 1= Disagree Strongly, 2= Disagree Moderately, 3= Disagree Slightly, 4=Neutral, 5= Agree Slightly, 6= Agree Moderately, 7= Agree Strongly.

**Table 2:** Level of understanding of interviewing concepts*

**Question Group**	**Pre-course**	**Post-course**	**Mean Gain**	***P* Value**#
Structure of the interview(6 items)	6.2	8.7	2.5	0.003
with the patient(6 items)Relationship with the patient(6 items)	6.5	8.4	1.9	0.002
Assessing affect(3 items)	6.6	8.6	2.0	0.001
Collecting data(6 items)	5.6	8.5	2.9	0.002

^*^ Each question group consists of 5 or 6 questions, each scored on a scale from 1(no understanding) to 10 (complete understanding). Presented are the mean scores of the students in each question group.

^#^ Using paired t test

**Figure 3:** Interviewing experience vs. baseline interviewing knowledge

The course was offered to students between their first and second year at 2 medical schools to benefit from the inter-institutional learning facilitated by Web-based distance education. Our enrollment target of 10 students was reached with 7 students from Boston University and 3 from the University of Massachusetts. Two working groups of 5 students were created, in our experience an ideal size for online course discussions [6,10].

## Results

Of the 10 students who started the course, one student dropped out of the course due to schedule conflicts, while 9 students completed the course and evaluations. Students made an average of 14 written postings during the 4-week course.

A qualitative analysis of the postings from course assignments consistently provided evidence of concept acquisition. A representative posting:

I realized that I never truly noticed any of Mary's or Ed's affect or non-verbal cues when I previously viewed the interview. However, when I watched the interview for a second time, I noticed many interactions that I had not before.

Major themes to emerge from the focus group are presented in Textbox 1.

**Major Themes**

Theme 1: Theoretical understanding and self-awareness.

Student: "I really do think I have a more organized picture in my head of what I want to do the next time I sit down."

Theme 2: Benefits compared to face-to-face interaction.

Student 1: "You're forced to think through a good response and good interpretation . . . "

Student 2: "I think it's great. I never thought that I would, I'm very computer illiterate, I never thought that I would choose to do something online as opposed to just on paper or in class, but it was so convenient and so like relaxing you know? I took away a ton from it too. I mean I really feel like I did."

Student 3: "You're so much more likely to learn if you're doing it when you're ready for it."

Student 4: "I felt like there were some things that I was really able to take my time with and understand."

Also apparent from the group was a desire for variation in interviews to analyze (Theme 3) and opportunities to apply the concepts to real patients (Theme 4).

Open-ended comments on the course evaluation form supported these themes, and provided more detail about advantages of online learning in this course over more conventional methods. Two students provided representative viewpoints:

Student 1: ". . . interacting with students in the on-line format allowed for well thought-out, comprehensive responses and much more insightful comments than sometimes heard in a classroom. I attribute this to the time one has to sit and think through a response, choose the words carefully, and elaborate uninterrupted. There's less pressure on-line, so you can piece together your thoughts with less stress and greater sincerity."

Student 2: "The strengths are the high level of participation and interaction and conversation (more so than in any other course so far.)"

### Quantitative Results

Students rated all aspects of the course highly (Table 1) and knowledge scores increased significantly ( *P*< .01) at the end of the course (Table 2). As can be expected, students who reported the least baseline knowledge reported the greatest increase in understanding of course concepts ( *r*= 0.79, *P*= .015) (Figure 4).

**Figure 4:** Gain in knowledge, by baseline knowledge

**Figure 5:** Correlation between words posted to course discussion group and gain in knowledge

Also, gain correlated with the number of words posted to the course discussion group ( *r*= 0.72, *P*=.02), suggesting that greater educational effort was correlated with greater self-reported gain in knowledge (Figure 5).

## Discussion

The students completing this course participated at a high level and rated it highly on learning process and achievement of course goals. Our data suggests that it increased student understanding of basic concepts underlying effective clinical communication. The course's acceptance was in large part due to its congruence with principles of adult learning [11] such as self pacing, reflective learning, and collaborative learning from peers [11]. Participants noted a number of advantages of online learning. Although there are only rare examples in the literature of online courses on communication skills for medical professionals or students [12], this study does add to the growing literature in medicine and in fields outside of medicine [13- 15], suggesting the effectiveness of Internet-based distance education. However, more-rigorous evaluations with control groups and a larger number of participants are required to establish which factors and participant characteristics are determinants of effective learning. Medical-education studies generally show that Internet-based instruction is at least as effective as conventional methods [16- 18] and in some cases superior [19- 21]. However, a recent meta-analysis of Web-based learning in medical education did not find this method superior to conventional methods, but did acknowledge that studies are needed that better compare instructional *methods* rather than comparing instructional *media*, as has been the focus of many studies to date, rendering conclusions about the relative merits of online vs face-to-face methods difficult to make [22]. A carefully-designed, carefully-taught, and carefully-evaluated online course may effect better learning outcomes than face to face instruction [23]. Based on the limitations of research to date, it is clear that further work is needed to assess the impact and acceptance of small group online education, and the role of faculty or other moderators in online medical education courses [19,24- 26]. The acceptance of this method in a broader, unselected student population will be of interest. Evidence suggests that most learners will ultimately be successful online learners [27]. We also note that self report of learning is less reliable than direct measurement of knowledge acquisition. However, there was consistency of findings from the mixed-method approach used to evaluate this course. Given the favorable results from this elective, we plan to integrate elements of this online course into the preclinical-years' communication-skills curricula for use by all first-year students.

Jordan Cohen, the President of the Association of American Medical Colleges (AAMC), in a speech once exhorted medical educators to seize "the potential of the technological revolution to transform the way students learn" [28]. In response, we have developed a new method of introducing the cognitive basis for communication using electronic technology. It should also be applicable to other content areas and is likely to prove particularly useful as medical education becomes increasingly decentralized.
