# Supplementary figures and images for: Collaborative e-Learning Using Streaming Video and Asynchronous Discussion Boards to Teach the Cognitive Foundation of Medical Interviewing: A Case Study
Source: J Med Internet Res. 2003 Jun 27;5(2):e13. doi: 10.2196/jmir.5.2.e13 (PMC1550556; doi:10.2196/jmir.5.2.e13)

## Slide 1
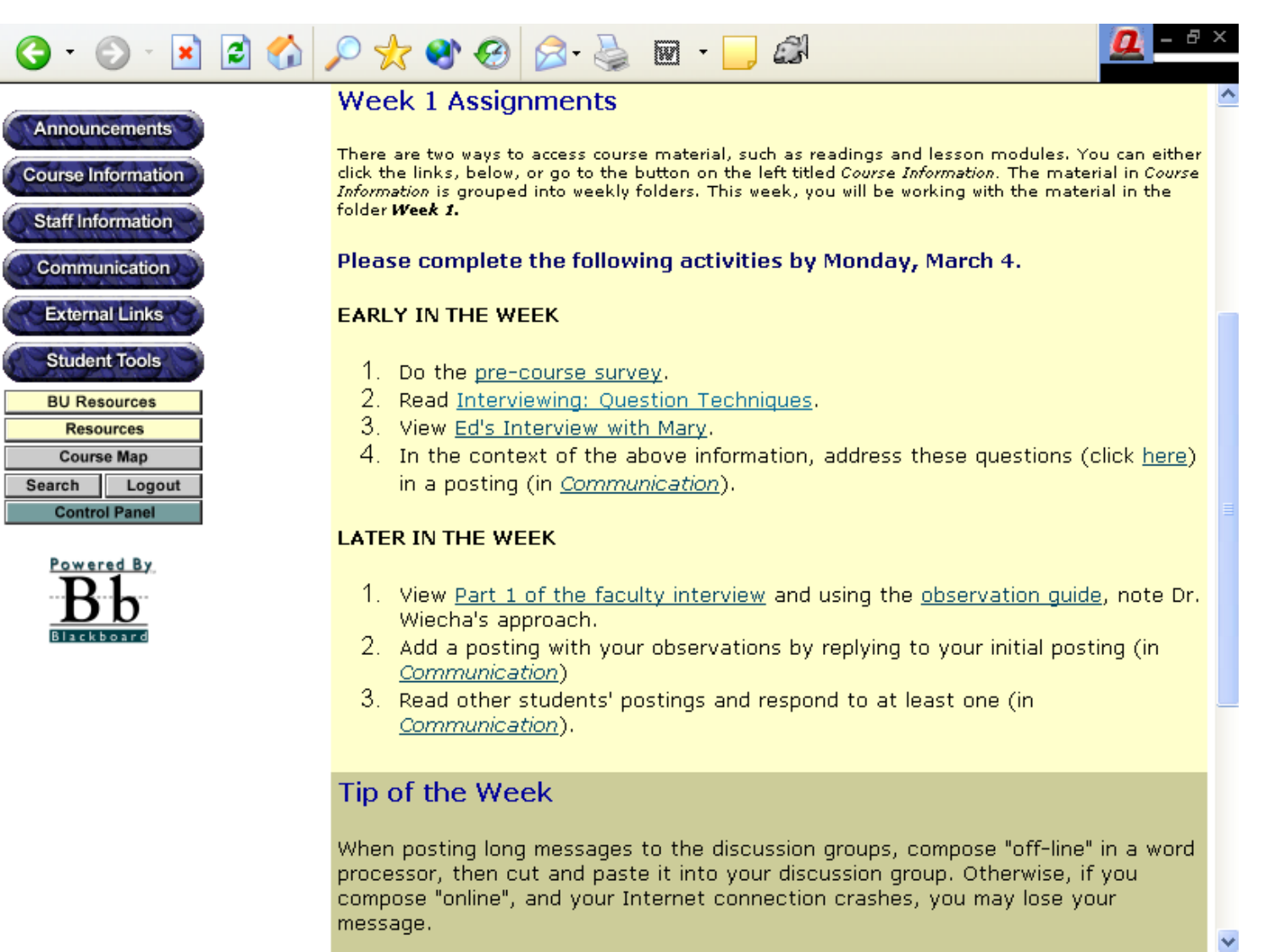

## Slide 2
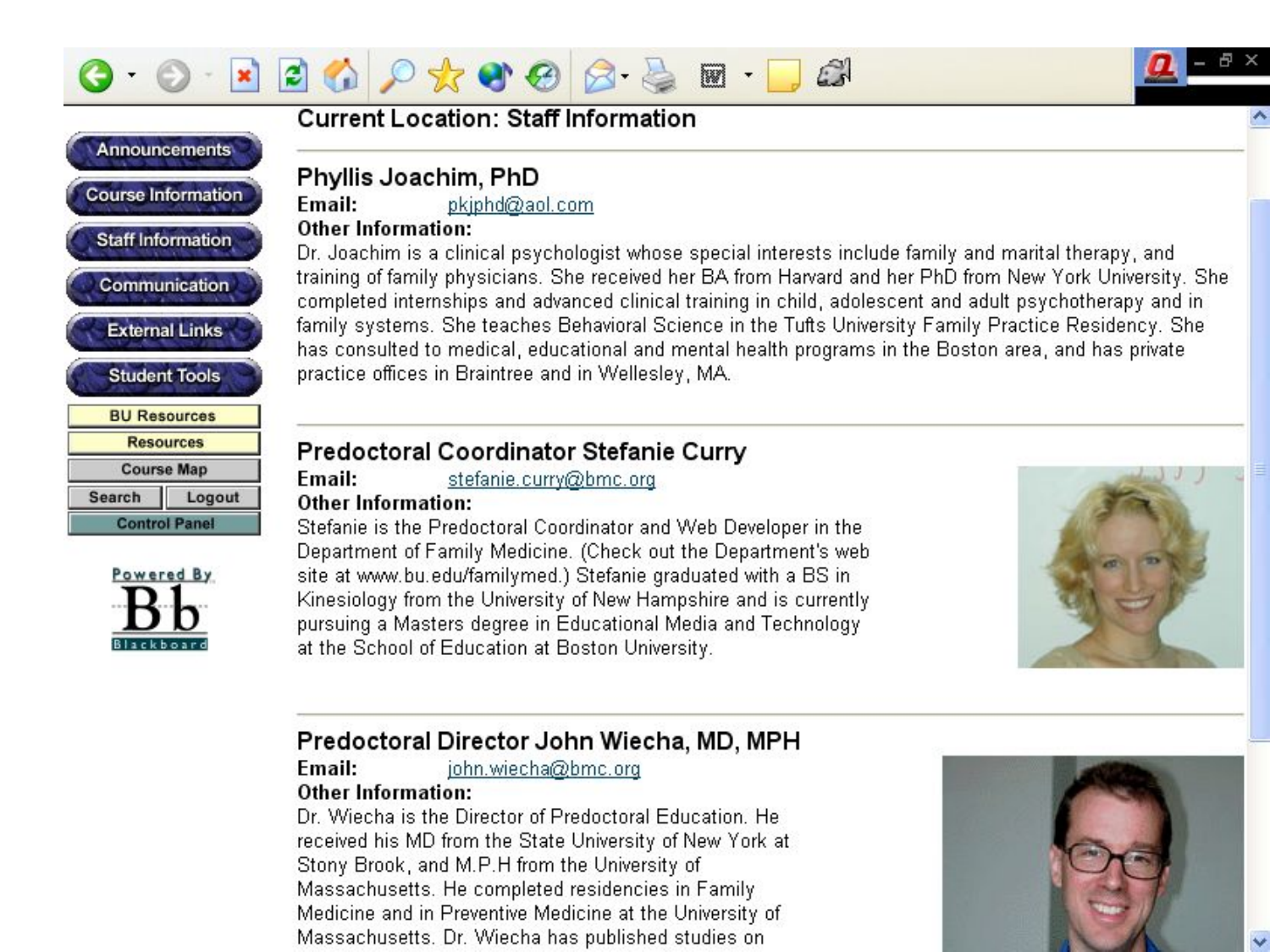

## Slide 3
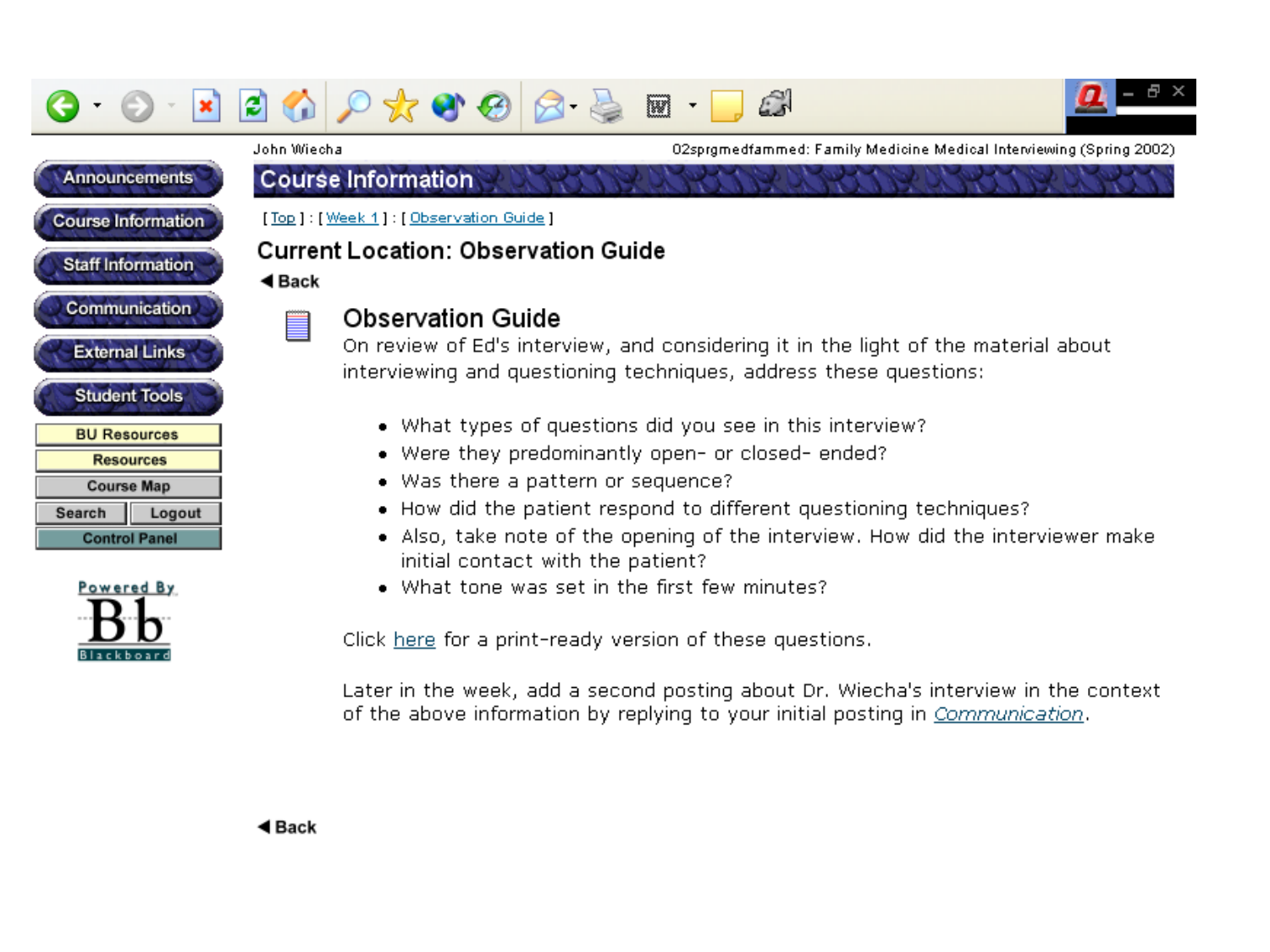

## Slide 4
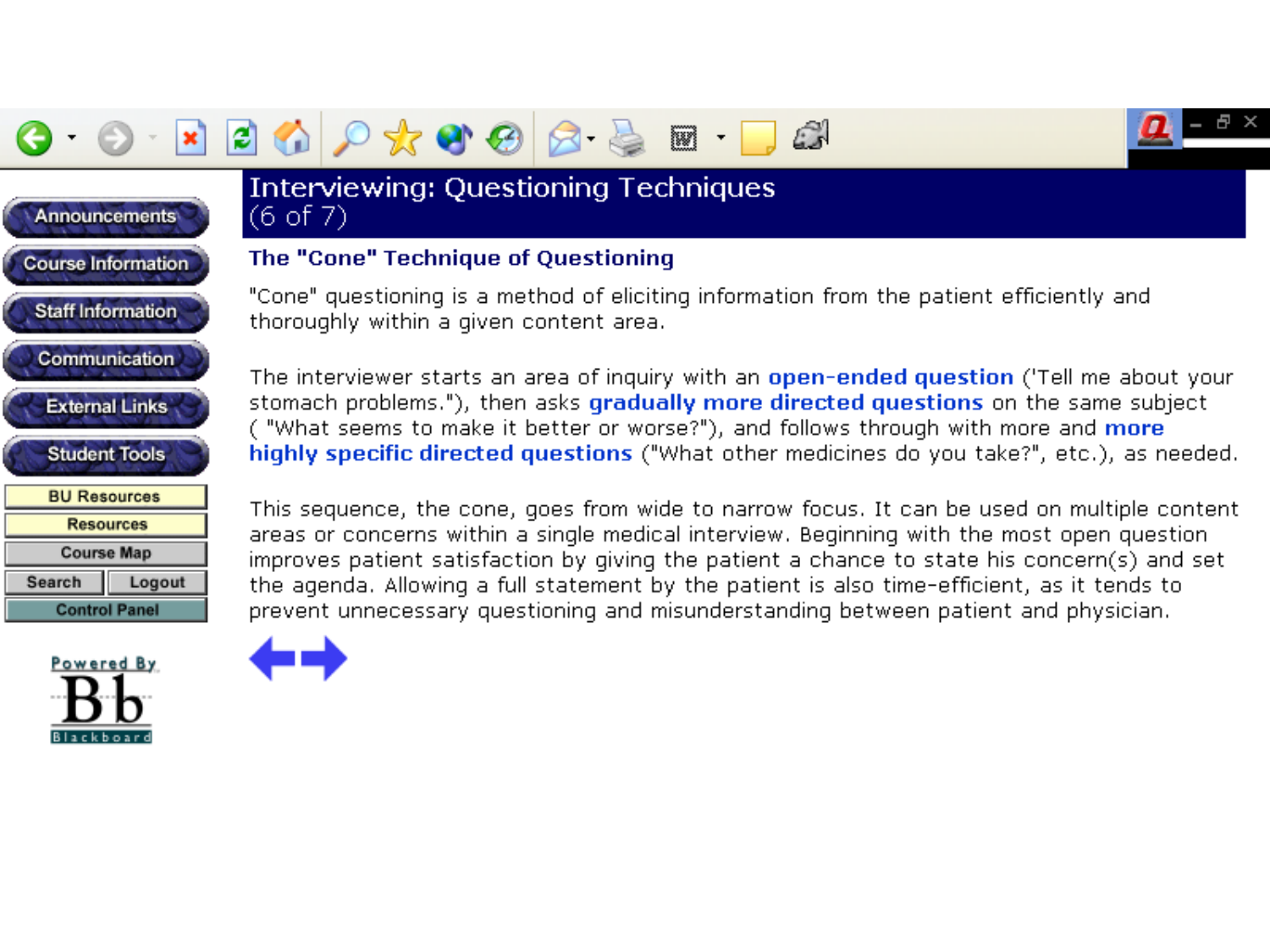

## Slide 5
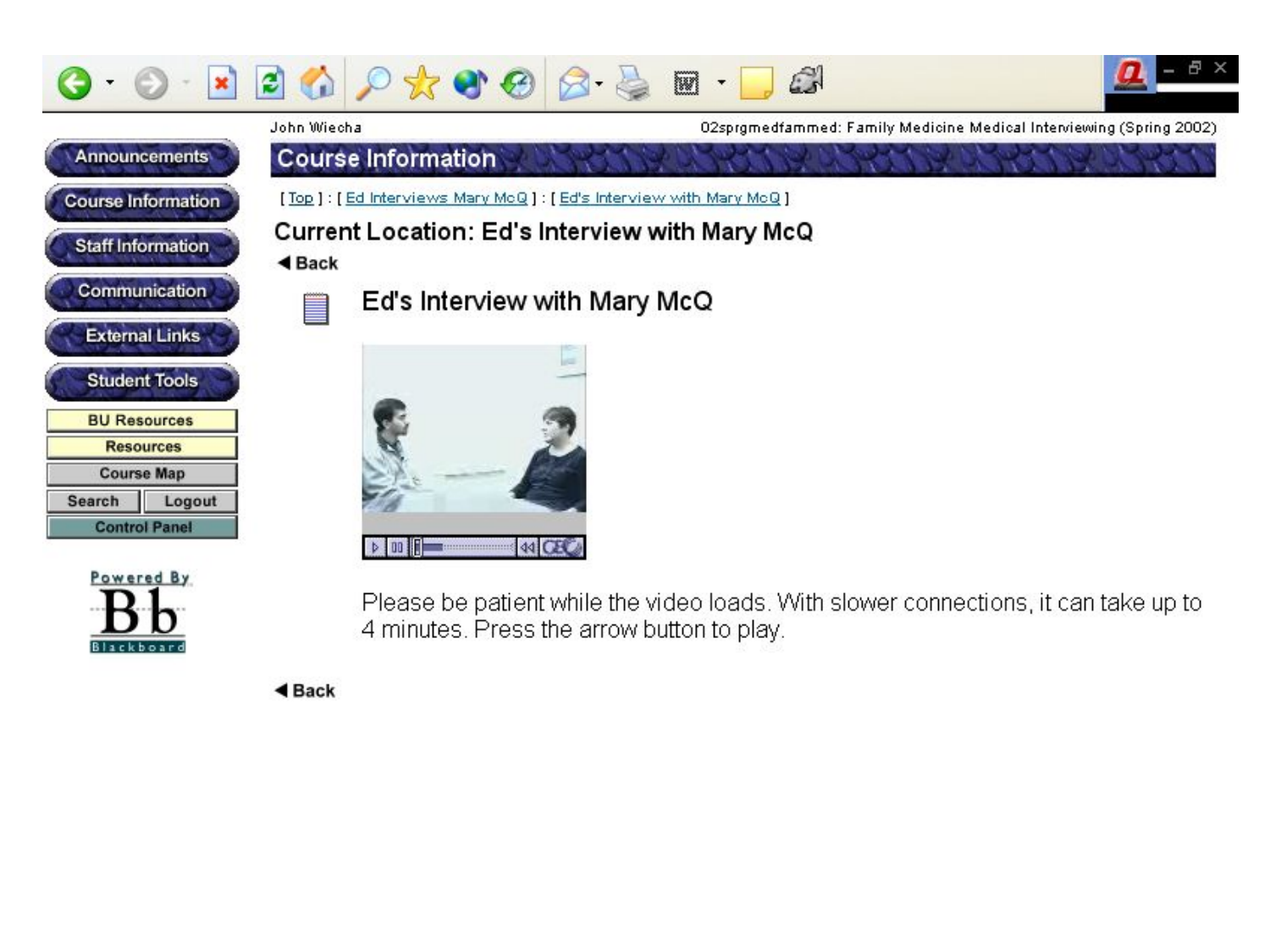

## Slide 6
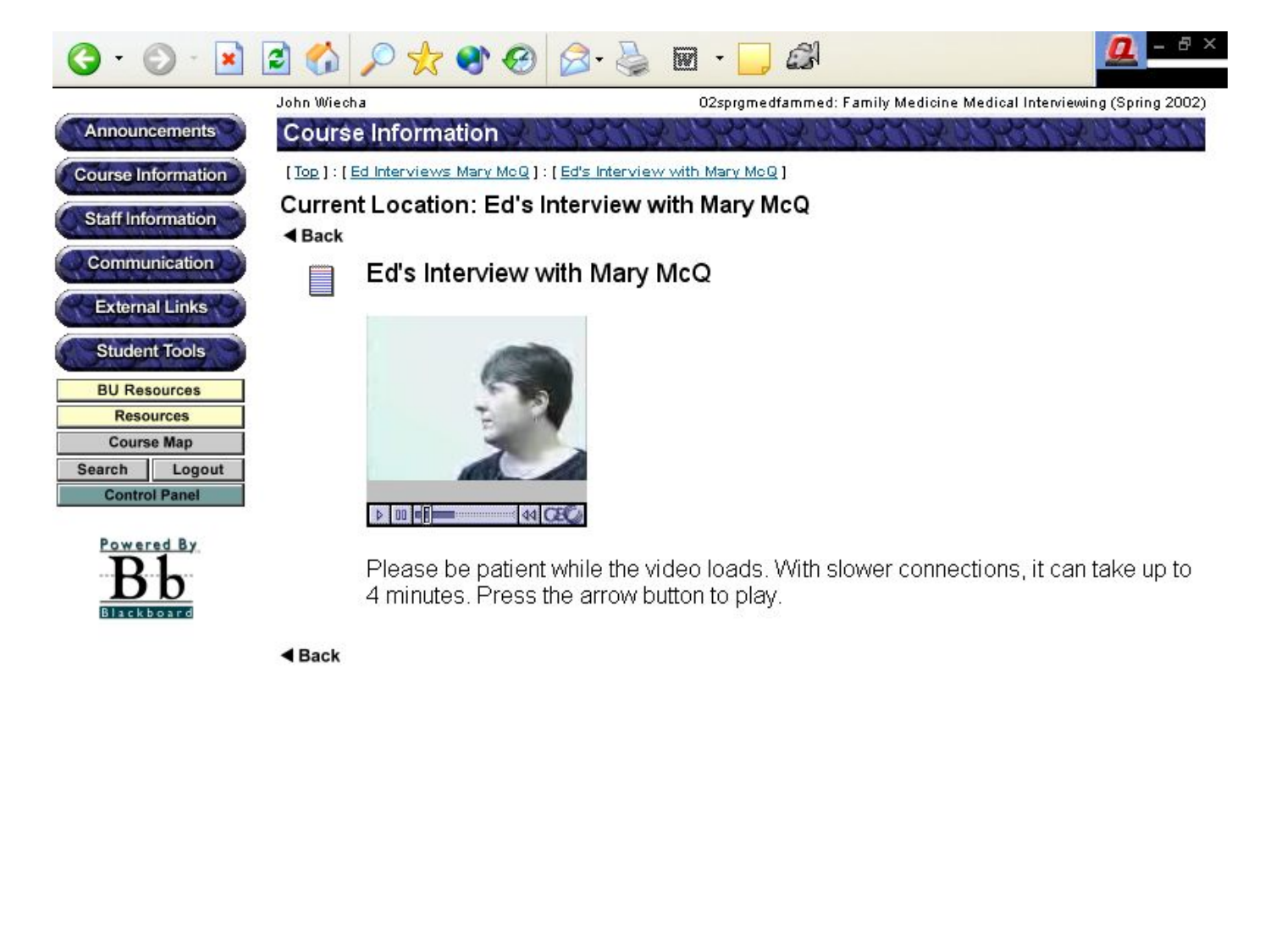

## Slide 7
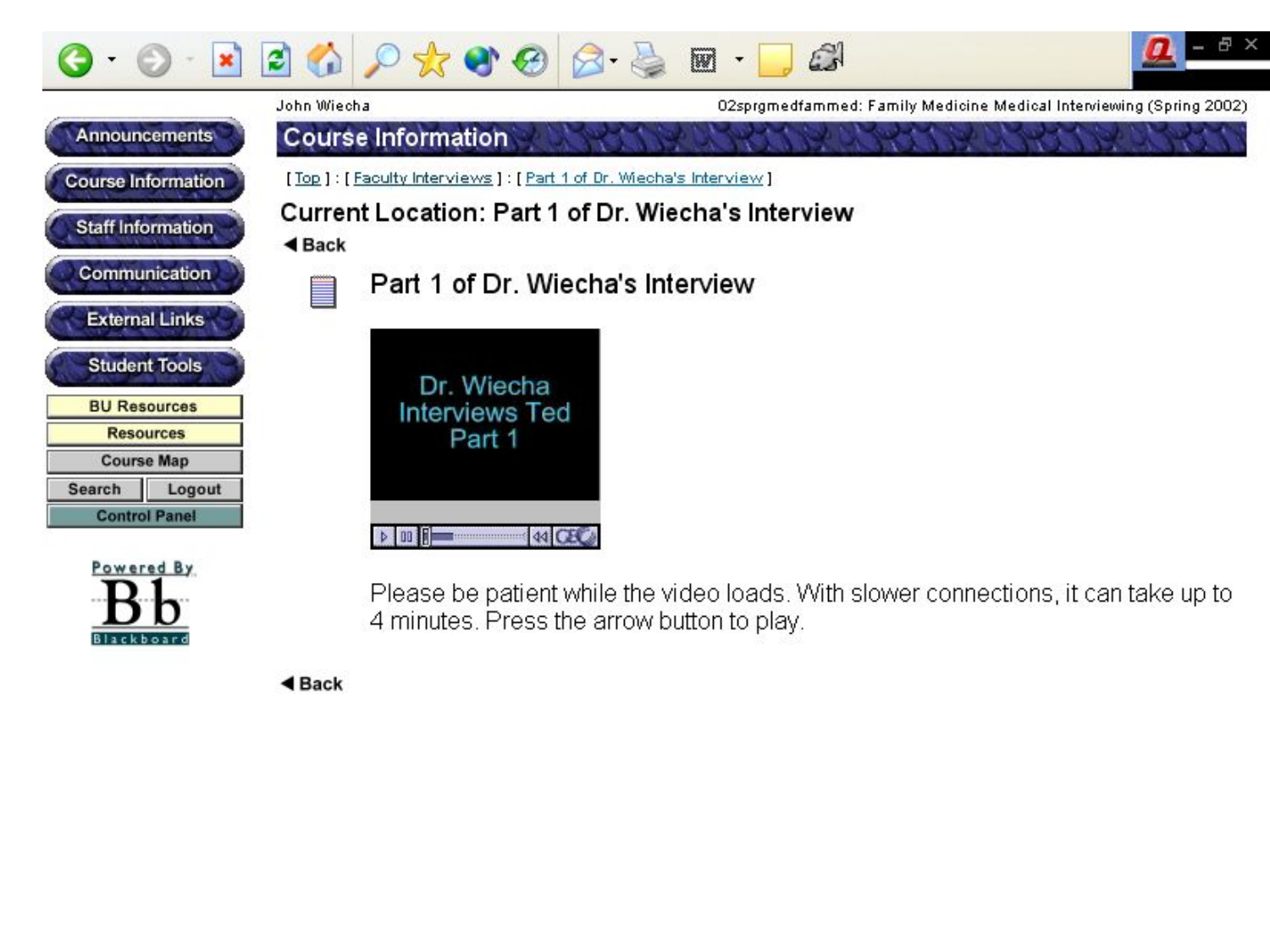

## Slide 8
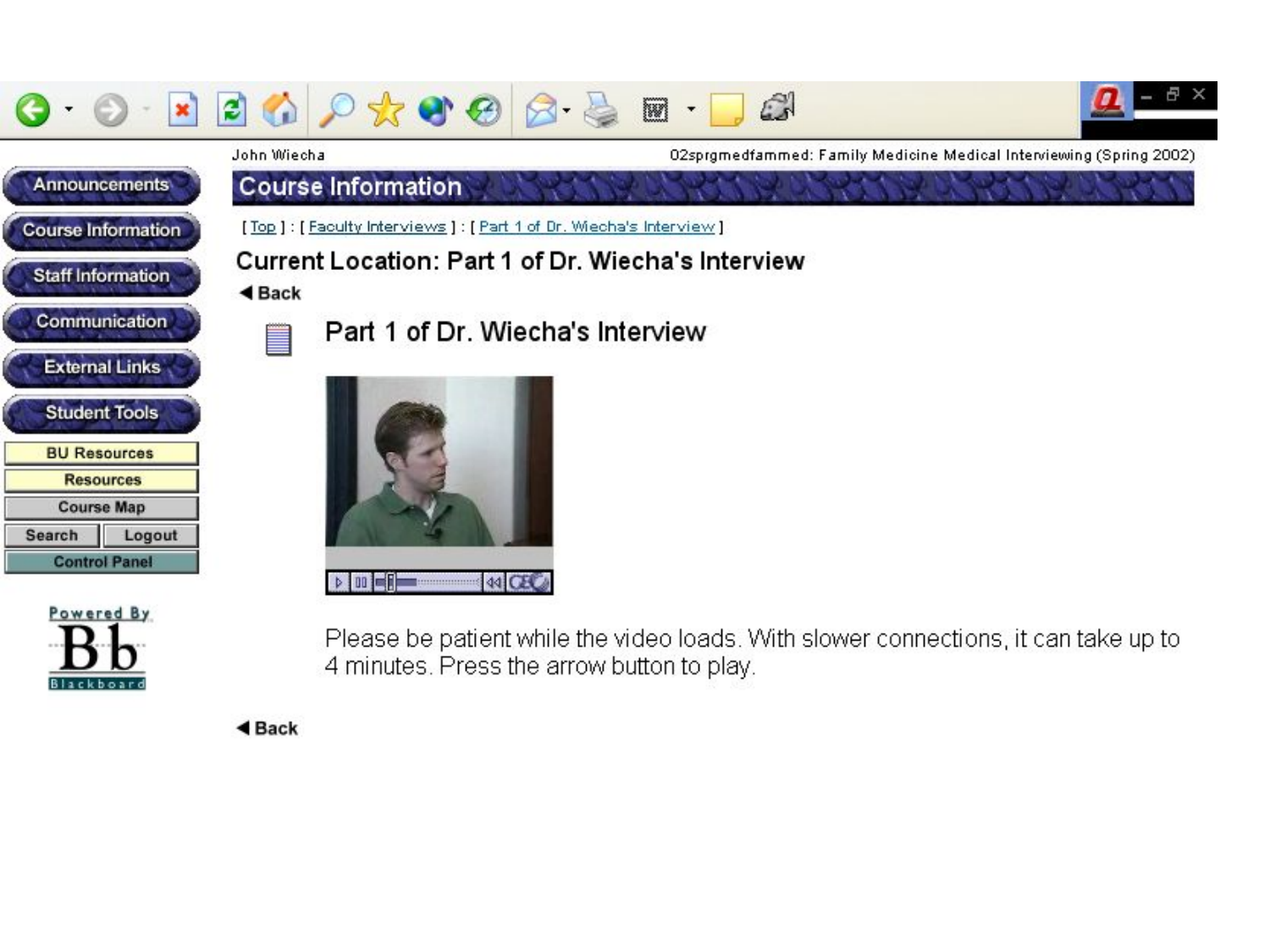

## Slide 9
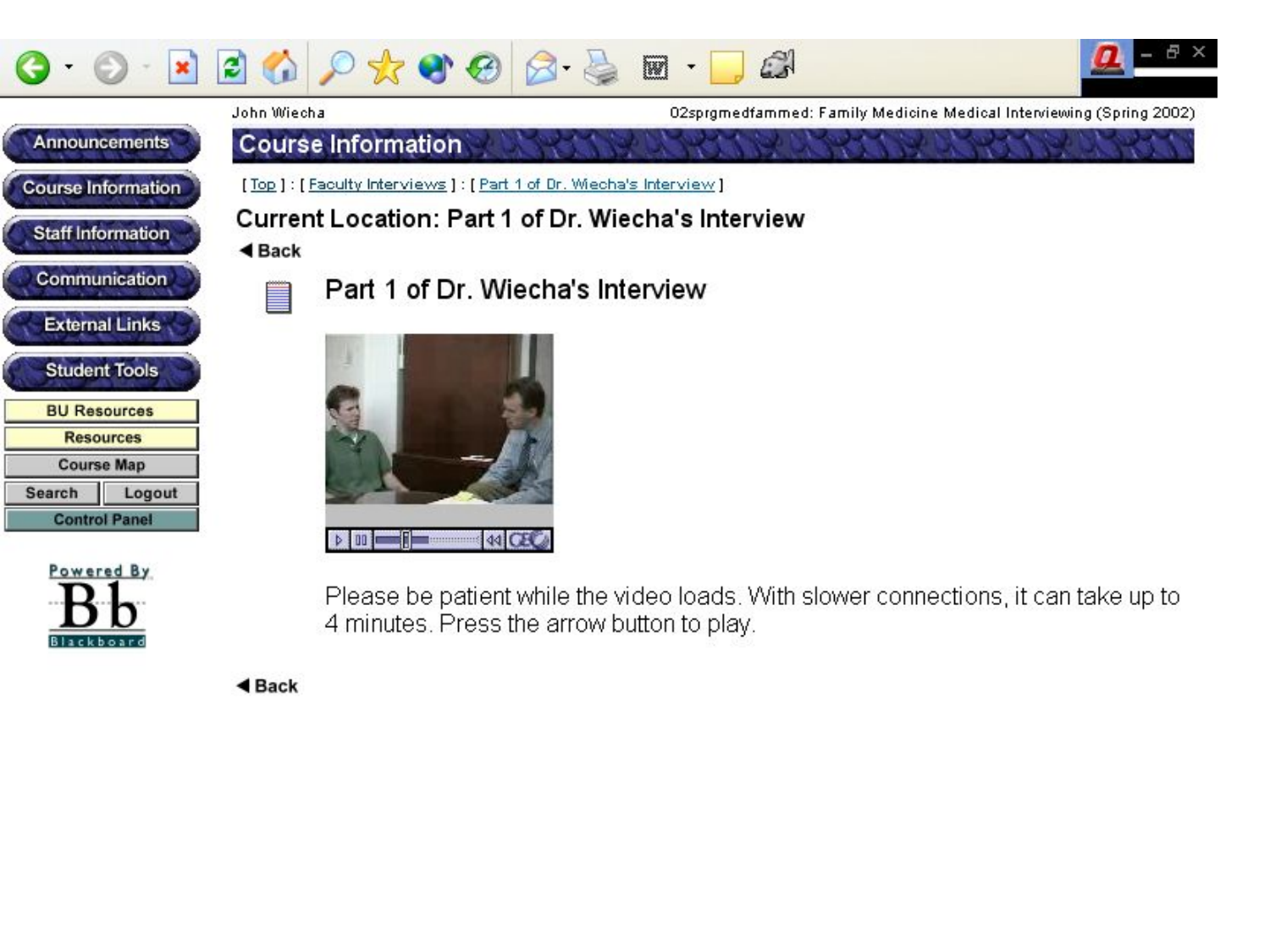

## Slide 10
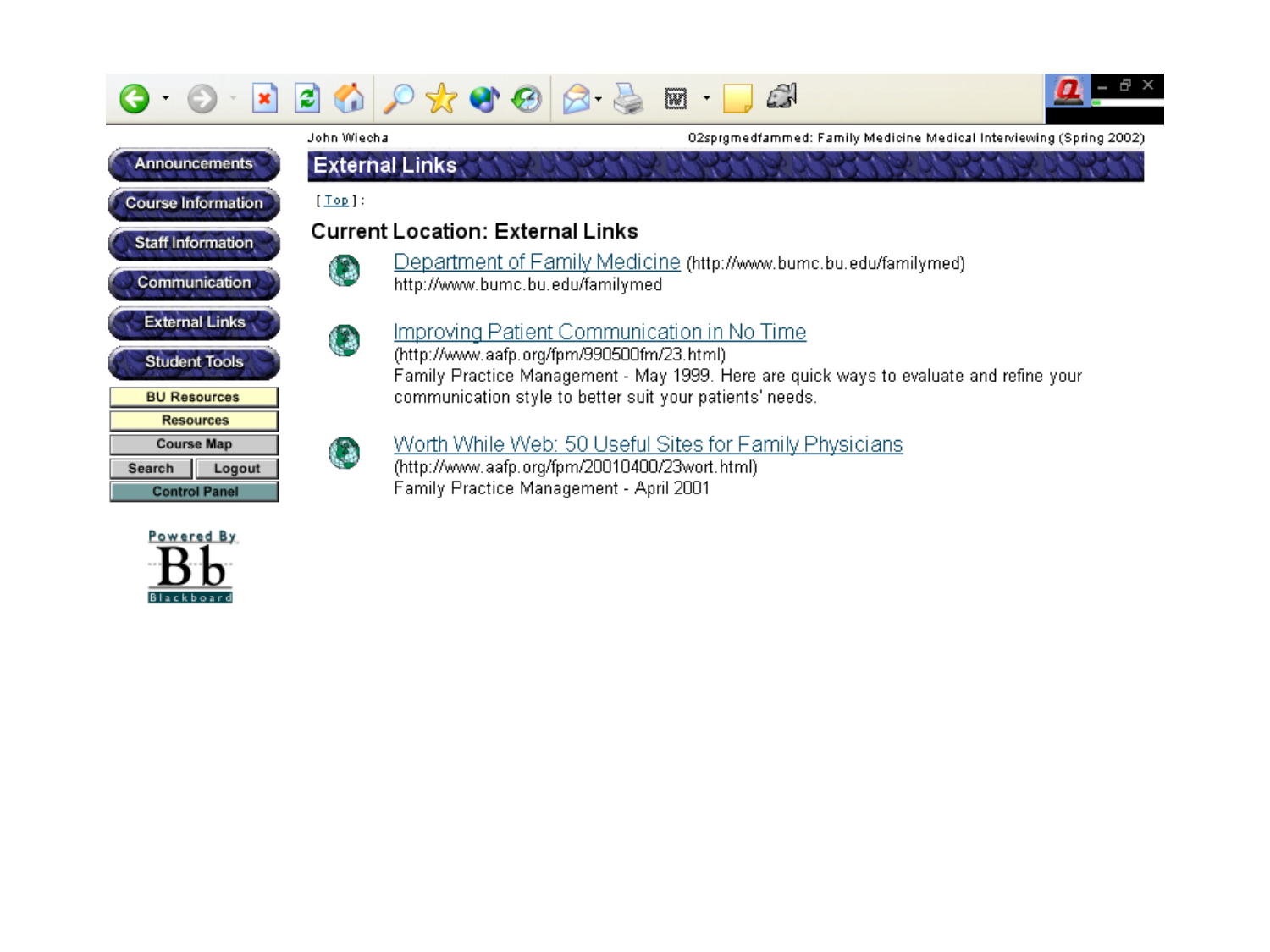

## Slide 11
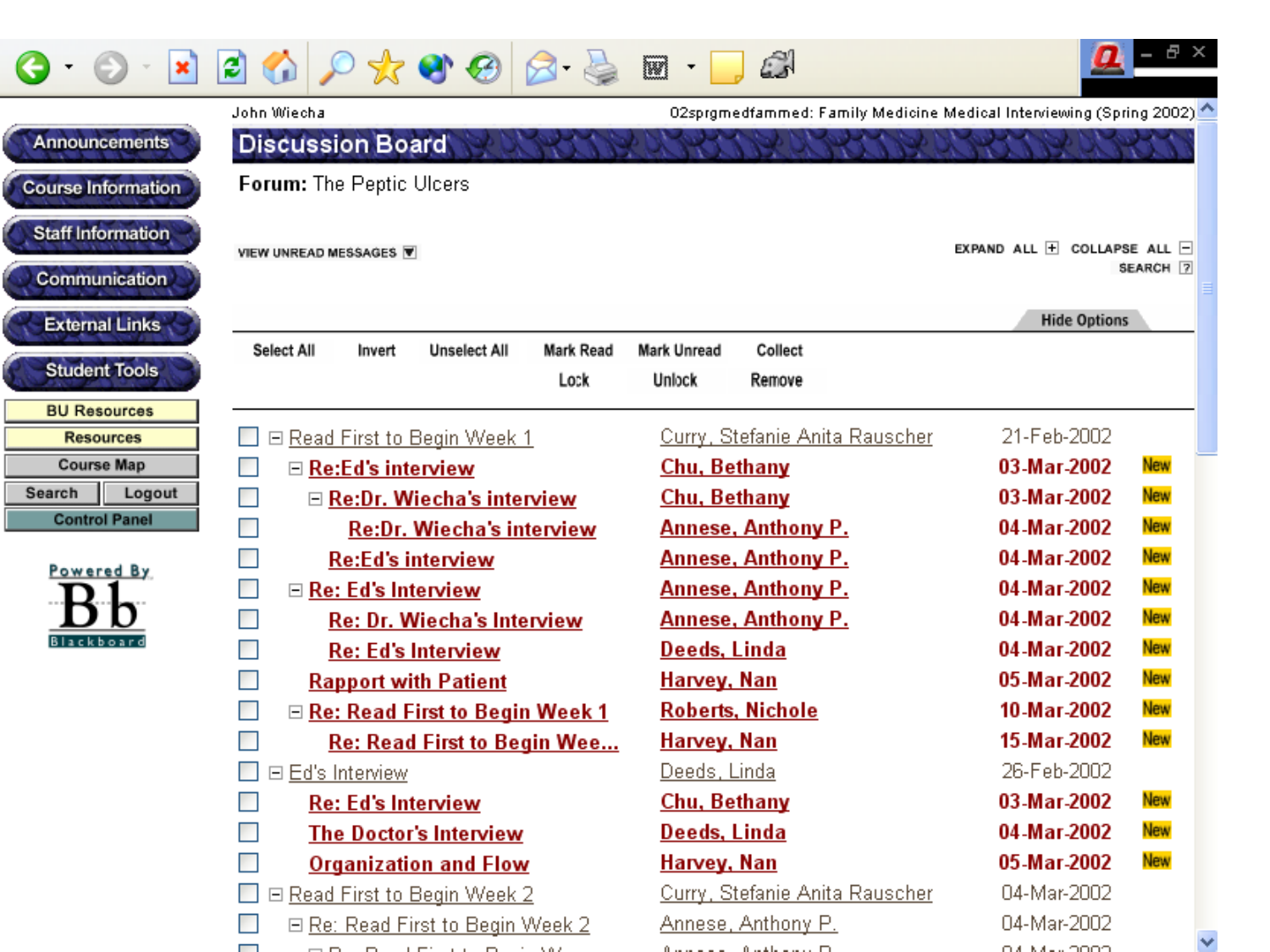

## Slide 12
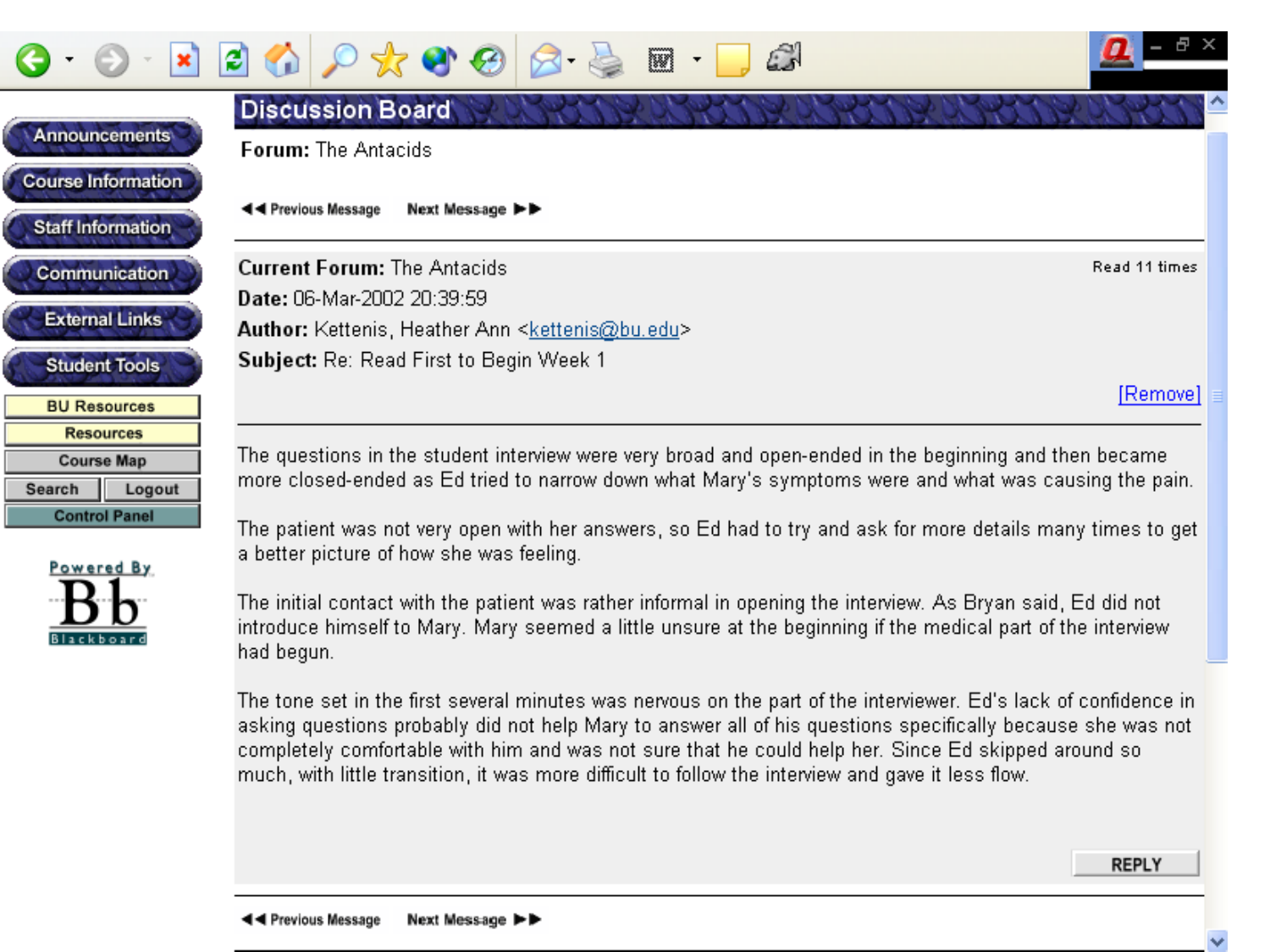

## Slide 13
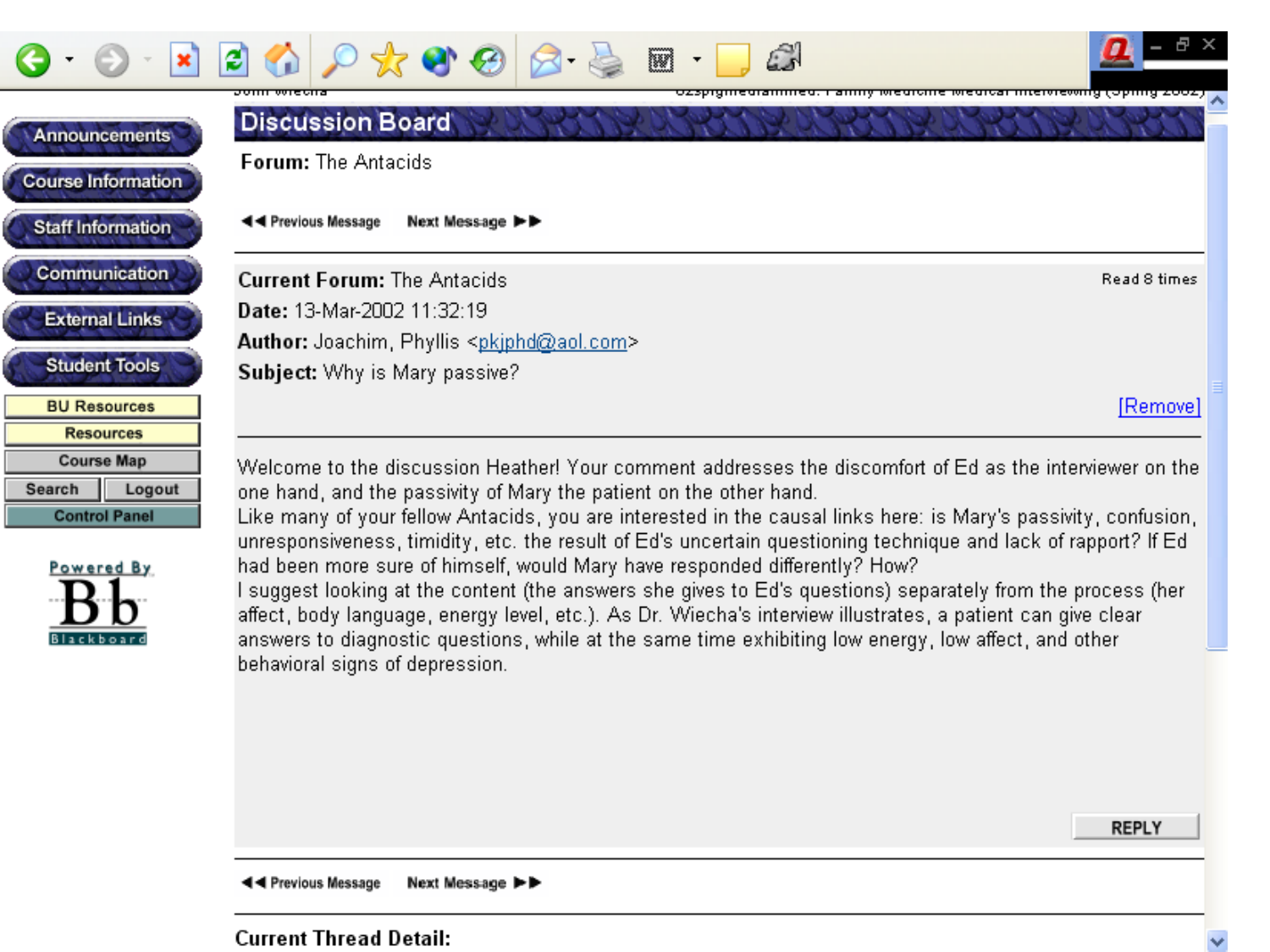

Supplement: Supplementary file 1 [file jmir_v5i2e13_app1.ppt]
