# Supplementary material for: Collaborative e-Learning Using Streaming Video and Asynchronous Discussion Boards to Teach the Cognitive Foundation of Medical Interviewing: A Case Study
Source: J Med Internet Res. 2003 Jun 27;5(2):e13. doi: 10.2196/jmir.5.2.e13 (PMC1550556; doi:10.2196/jmir.5.2.e13)
Supplement: Supplementary file 2 [file jmir_v5i2e13_app2.html]

Interviewing Course Pre Survey


**Boston
University School of Medicine**  
Family Medicine Medical Interviewing Online Course  
 Pre Survey - Spring 2002

---

Please
help us improve this online course by answering the following questions.
  
Click "Submit Survey" when you are finished.

**Part
I**

|  |  |  |  |  |
| --- | --- | --- | --- | --- |
| **First Name:** |  | | | |
| **Last Name:** |  | | | |
| **E-mail Address:** |  | | | |

**Part
II**

|  |  |  |
| --- | --- | --- |
| 1. Do you own a computer? | Yes | No |
| 2. Do you have Internet access at home? | Yes | No |
| 3. Have you ever taken an online course? | Yes | No |
| 4. Number of hours you spend online in an average week: | hours | |
| 5. Did you participate in the Family Medicine sub-option in the first year Introduction to Clinical Medicine Course? | Yes | No |
| 6. Approximate number of patients interviewed/histories taken (including standardized patients) to date: |  | |

**Part
III**

|  |  |  |  |  |  |  |  |
| --- | --- | --- | --- | --- | --- | --- | --- |
| ***Please indicate to what extent you agree or disagree with each of the following statements:*** | Disagree Strongly | Disagree Moderately | Disagree Slightly | Neutral | Agree Slightly | Agree Moderately | Agree Strongly |
| 7. The Internet is a useful tool for learning medicine. |  |  |  |  |  |  |  |
| 8. An online course is an effective method for learning the principles of patient interviewing. |  |  |  |  |  |  |  |
| 9. Learning the concepts underlying effective interviewing can improve my interviewing skills. |  |  |  |  |  |  |  |
| 10. This course will help me improve my interviewing skills. |  |  |  |  |  |  |  |

**Part
IV**

|  |  |  |  |  |  |  |  |  |  |  |
| --- | --- | --- | --- | --- | --- | --- | --- | --- | --- | --- |
|  | None(1)...........................................................Complete(10) | | | | | | | | | |
| ***Please rate your level of understanding of each of the following concepts:*** | 1 | 2 | 3 | 4 | 5 | 6 | 7 | 8 | 9 | 10 |
|
| 11. Initiating the medical interview. |  |  |  |  |  |  |  |  |  |  |
| 12. Developing rapport with the patient during the interview. |  |  |  |  |  |  |  |  |  |  |
| 13. Observing patient interactions during the interview. |  |  |  |  |  |  |  |  |  |  |
| 14. Eliciting information during the interview. |  |  |  |  |  |  |  |  |  |  |
| 15. Maintaining control and focus in the interview. |  |  |  |  |  |  |  |  |  |  |
| 16. Awareness of the range of different questioning techniques. |  |  |  |  |  |  |  |  |  |  |
| 17. Understanding how and when to use different questioning techniques in the medical interview. |  |  |  |  |  |  |  |  |  |  |
| 18. Purpose of the opening part of the interview. |  |  |  |  |  |  |  |  |  |  |
| 19. The social phase of the interview. |  |  |  |  |  |  |  |  |  |  |
| 20. Setting of expectations in the interview. |  |  |  |  |  |  |  |  |  |  |
|  | None(1).....................................................Complete(10) | | | | | | | | | |
| ***Please rate your level of understanding of each of the following concepts:*** | 1 | 2 | 3 | 4 | 5 | 6 | 7 | 8 | 9 | 10 |
|
| 21. Negotiation or limit setting with the patient. |  |  |  |  |  |  |  |  |  |  |
| 22. Use of connecting statements in transition. |  |  |  |  |  |  |  |  |  |  |
| 23. Labeling of subjects covered. |  |  |  |  |  |  |  |  |  |  |
| 24. Objectives of the closure in the interview. |  |  |  |  |  |  |  |  |  |  |
| 25. The affective state of the patient. |  |  |  |  |  |  |  |  |  |  |
| 26. Non-verbal interview cues. |  |  |  |  |  |  |  |  |  |  |
| 27. Use of empathic responses to patients during the interview. |  |  |  |  |  |  |  |  |  |  |
| 28. Regulating the flow of the interview. |  |  |  |  |  |  |  |  |  |  |
| 29. Bringing closure to the interview. |  |  |  |  |  |  |  |  |  |  |
| 30. Observational Skills (watching, listening). |  |  |  |  |  |  |  |  |  |  |
| 31. The cardinal features defining a symptom. |  |  |  |  |  |  |  |  |  |  |
|  | Novice(1)....................................................Advanced(10) | | | | | | | | | |
|  | 1 | 2 | 3 | 4 | 5 | 6 | 7 | 8 | 9 | 10 |
|
| 32. Please rate your medical interviewing skills. |  |  |  |  |  |  |  |  |  |  |
