# Supplementary material for: Collaborative e-Learning Using Streaming Video and Asynchronous Discussion Boards to Teach the Cognitive Foundation of Medical Interviewing: A Case Study
Source: J Med Internet Res. 2003 Jun 27;5(2):e13. doi: 10.2196/jmir.5.2.e13 (PMC1550556; doi:10.2196/jmir.5.2.e13)
Supplement: Supplementary file 3 [file jmir_v5i2e13_app3.html]

Interviewing Course Post Survey


**Boston
University School of Medicine**  
Family Medicine Medical Interviewing  
Online Course  
 Post Survey - Spring 2002

---


Please
help us improve this online course by answering the following questions.   
Click "Submit Survey" when you are finished.

**Part
I**

|  |  |  |  |  |
| --- | --- | --- | --- | --- |
| **First Name:** |  | | | |
| **Last Name:** |  | | | |
| **E-mail Address:** |  | | | |

**Part
II**

|  |  |  |  |  |  |  |
| --- | --- | --- | --- | --- | --- | --- |
| **How well did video function on the computer you used for this course?** | Very well Well Fairly well Not well at all | | | | | |
| **Did you use a dial-up connection?** | Yes No | | | | | |

**Part
III** (Questions 7-10)

(Note:
Questions start at #7; there are no questions #1-6.)

|  |  |  |  |  |  |  |  |
| --- | --- | --- | --- | --- | --- | --- | --- |
| ***Please indicate to what extent you agree or disagree with each of the following statements:*** | Disagree Strongly | Disagree Moderately | Disagree Slightly | Neutral | Agree Slightly | Agree Moderately | Agree Strongly |
| 7. The Internet is a useful tool for learning medicine. |  |  |  |  |  |  |  |
| 8. An online course is an effective method for learning the principles of patient interviewing. |  |  |  |  |  |  |  |
| 9. Learning the concepts underlying effective interviewing can improve my interviewing skills. |  |  |  |  |  |  |  |
| 10. This course will help me improve my interviewing skills. |  |  |  |  |  |  |  |

**Part
IV** (Questions 11-32)

|  |  |  |  |  |  |  |  |  |  |  |
| --- | --- | --- | --- | --- | --- | --- | --- | --- | --- | --- |
| ***Please rate your level of understanding of each of the following concepts:*** | (**1**) No Understanding to (**10**) Complete Understanding | | | | | | | | | |
|  | 1 | 2 | 3 | 4 | 5 | 6 | 7 | 8 | 9 | 10 |
|  |  |  |  |  |  |  |  |  |  |  |
| 11. Initiating the medical interview. |  |  |  |  |  |  |  |  |  |  |
| 12. Developing rapport with the patient during the interview. |  |  |  |  |  |  |  |  |  |  |
| 13. Observing patient interactions during the interview. |  |  |  |  |  |  |  |  |  |  |
| 14. Eliciting information during the interview. |  |  |  |  |  |  |  |  |  |  |
| 15. Maintaining control and focus in the interview. |  |  |  |  |  |  |  |  |  |  |
| 16. Awareness of the range of different questioning techniques. |  |  |  |  |  |  |  |  |  |  |
| 17. Understanding how and when to use different questioning techniques in the medical interview. |  |  |  |  |  |  |  |  |  |  |
| 18. Purpose of the opening part of the interview. |  |  |  |  |  |  |  |  |  |  |
| 19. The social phase of the interview. |  |  |  |  |  |  |  |  |  |  |
| 20. Setting of expectations in the interview. |  |  |  |  |  |  |  |  |  |  |
| ***Please rate your level of understanding of each of the following concepts:*** | (**1**) No Understanding to (**10**) Complete Understanding | | | | | | | | | |
|  | 1 | 2 | 3 | 4 | 5 | 6 | 7 | 8 | 9 | 10 |
|  |  |  |  |  |  |  |  |  |  |  |
| 21. Negotiation or limit setting with the patient. |  |  |  |  |  |  |  |  |  |  |
| 22. Use of connecting statements in transition. |  |  |  |  |  |  |  |  |  |  |
| 23. Labeling of subjects covered. |  |  |  |  |  |  |  |  |  |  |
| 24. Objectives of the closure in the interview. |  |  |  |  |  |  |  |  |  |  |
| 25. The affective state of the patient. |  |  |  |  |  |  |  |  |  |  |
| 26. Nonverbal interview cues. |  |  |  |  |  |  |  |  |  |  |
| 27. Use of empathic responses to patients during the interview. |  |  |  |  |  |  |  |  |  |  |
| 28. Regulating the flow of the interview. |  |  |  |  |  |  |  |  |  |  |
| 29. Bringing closure to the interview. |  |  |  |  |  |  |  |  |  |  |
| 30. Observational Skills (watching, listening). |  |  |  |  |  |  |  |  |  |  |
| 31. The cardinal features defining a symptom. |  |  |  |  |  |  |  |  |  |  |
|  | (**1**) Novice to (**10**) Advanced | | | | | | | | | |
|  | 1 | 2 | 3 | 4 | 5 | 6 | 7 | 8 | 9 | 10 |
|  |  |  |  |  |  |  |  |  |  |  |
| 32. Please rate your medical interviewing skills. |  |  |  |  |  |  |  |  |  |  |

**Part
V** (Questions 33-55)

***Next,
questions about the ONLINE INTERVIEWING COURSE:***

|  |  |  |  |  |  |  |  |
| --- | --- | --- | --- | --- | --- | --- | --- |
| ***Questions regarding your experience with the ONLINE INTERVIEWING COURSE:*** | Disagree Strongly | Disagree Moderately | Disagree Slightly | Neutral | Agree Slightly | Agree Moderately | Agree Strongly |
| 33. During this course I learned from other students. |  |  |  |  |  |  |  |
| 34. I received feedback on my questions and concerns from BU faculty during the course. |  |  |  |  |  |  |  |
| 35. I enjoyed the online course. |  |  |  |  |  |  |  |
| 36. I would like more online courses offered. |  |  |  |  |  |  |  |
| ***Questions regarding your experience with the ONLINE INTERVIEWING COURSE:*** | Disagree Strongly | Disagree Moderately | Disagree Slightly | Neutral | Agree Slightly | Agree Moderately | Agree Strongly |
| 37. Viewing videotapes of student interviews online helped me to improve my patient interviewing skills. |  |  |  |  |  |  |  |
| 38. The videotapes were effective in demonstrating principles of interviewing. |  |  |  |  |  |  |  |
| 39. The student interview was effective in demonstrating principles of interviewing. |  |  |  |  |  |  |  |
| ***Questions regarding your experience with the ONLINE INTERVIEWING COURSE:*** | Disagree Strongly | Disagree Moderately | Disagree Slightly | Neutral | Agree Slightly | Agree Moderately | Agree Strongly |
| 40. Viewing the faculty interview (Dr. Wiecha) was effective in demonstrating principles of interviewing. |  |  |  |  |  |  |  |
| 41. I valued interacting with other students online via the threaded discussion groups. |  |  |  |  |  |  |  |
| 42. I valued interacting with faculty online via the threaded discussions groups. |  |  |  |  |  |  |  |
| 43. The online course was easy to use. |  |  |  |  |  |  |  |
| ***Questions regarding your experience with the ONLINE INTERVIEWING COURSE:*** | Disagree Strongly | Disagree Moderately | Disagree Slightly | Neutral | Agree Slightly | Agree Moderately | Agree Strongly |
| 44. I had adequate time in my schedule to complete the assignments in the online course. |  |  |  |  |  |  |  |
| 45. I would recommend that other students take this course. |  |  |  |  |  |  |  |
| 46. I would be interested in other online courses in medical school. |  |  |  |  |  |  |  |
| 47. I would like to review the course material before reentering clinical work this fall. |  |  |  |  |  |  |  |

48.
I was engaged in patient care while doing this course **Yes
No**

|  |  |  |  |  |  |  |  |
| --- | --- | --- | --- | --- | --- | --- | --- |
| ***Answer Question 50 ONLY if you answered YES to Question 49*** | Disagree Strongly | Disagree Moderately | Disagree Slightly | Neutral | Agree Slightly | Agree Moderately | Agree Strongly |
| 49. I applied the material from this course during my clinical contact with patients. |  |  |  |  |  |  |  |

50. Usual
number of hours devoted to the Online Interviewing Course each week:
hours

51. Where
did you usually access the
Internet for this work?   

Office (1)
Home (2)
BU (3)
Other:

52. When
did you usually go online?

53.
I would be interested in taking an online elective in the following
topics:

Ethics
Literature in Medicine
Managed Care
History of Medicine
None

54.
What are the strengths of this course?

55.
Suggest some improvements for this course.

56.
How have you applied what you learned in the course to your real patient
encounters? Please give examples if you have some!

---

**Thank
you! Please click "Submit Survey".**
